# Staging investigations for oesophageal cancer: a meta-analysis

**DOI:** 10.1038/sj.bjc.6604200

**Published:** 2008-01-22

**Authors:** E P M van Vliet, M H Heijenbrok-Kal, M G M Hunink, E J Kuipers, P D Siersema

**Affiliations:** 1 Department of Gastroenterology and Hepatology, Erasmus MC – University Medical Center Rotterdam, Rotterdam, The Netherlands; 2 Department of Epidemiology and Biostatistics, Erasmus MC – University Medical Center Rotterdam, Rotterdam, The Netherlands; 3 Department of Radiology, Erasmus MC – University Medical Center Rotterdam, Rotterdam, The Netherlands; 4 Department of Internal Medicine, Erasmus MC – University Medical Center Rotterdam, Rotterdam, The Netherlands; 5 Department of Gastroenterology and Hepatology, University Medical Center Utrecht, Utrecht, The Netherlands

**Keywords:** meta-analysis, staging, oesophageal cancer

## Abstract

The aim of the study was to compare the diagnostic performance of endoscopic ultrasonography (EUS), computed tomography (CT), and ^18^F-fluoro-2-deoxy-D-glucose positron emission tomography (FDG-PET) in staging of oesophageal cancer. PubMed was searched to identify English-language articles published before January 2006 and reporting on diagnostic performance of EUS, CT, and/or FDG-PET in oesophageal cancer patients. Articles were included if absolute numbers of true-positive, false-negative, false-positive, and true-negative test results were available or derivable for regional, celiac, and abdominal lymph node metastases and/or distant metastases. Sensitivities and specificities were pooled using a random effects model. Summary receiver operating characteristic analysis was performed to study potential effects of study and patient characteristics. Random effects pooled sensitivities of EUS, CT, and FDG-PET for regional lymph node metastases were 0.80 (95% confidence interval 0.75–0.84), 0.50 (0.41–0.60), and 0.57 (0.43–0.70), respectively, and specificities were 0.70 (0.65–0.75), 0.83 (0.77–0.89), and 0.85 (0.76–0.95), respectively. Diagnostic performance did not differ significantly across these tests. For detection of celiac lymph node metastases by EUS, sensitivity and specificity were 0.85 (0.72–0.99) and 0.96 (0.92–1.00), respectively. For abdominal lymph node metastases by CT, these values were 0.42 (0.29–0.54) and 0.93 (0.86–1.00), respectively. For distant metastases, sensitivity and specificity were 0.71 (0.62–0.79) and 0.93 (0.89–0.97) for FDG-PET and 0.52 (0.33–0.71) and 0.91 (0.86–0.96) for CT, respectively. Diagnostic performance of FDG-PET for distant metastases was significantly higher than that of CT, which was not significantly affected by study and patient characteristics. The results suggest that EUS, CT, and FDG-PET each play a distinctive role in the detection of metastases in oesophageal cancer patients. For the detection of regional lymph node metastases, EUS is most sensitive, whereas CT and FDG-PET are more specific tests. For the evaluation of distant metastases, FDG-PET has probably a higher sensitivity than CT. Its combined use could however be of clinical value, with FDG-PET detecting possible metastases and CT confirming or excluding their presence and precisely determining the location(s).

To optimise the selection of patients with oesophageal cancer for a curative or palliative treatment option, it is important to determine the depth of infiltration of the tumour into the oesophageal wall (T stage), and the presence of malignant regional lymph nodes (N stage) and distant metastases (M stage). For N stage, N0 and N1 indicate the absence or presence of regional lymph node metastases, respectively. Similarly, M0 indicates the absence and M1 the presence of distant metastases (Fleming *et al*, 1997). Whether a malignant lymph node is defined as N1 or M1 depends on the location of the primary tumour. For example, malignant celiac lymph nodes are staged as M1a if the primary tumour is located in the distal part of the oesophagus, but as stage M1b if the tumour is located in the more proximal part of the oesophagus and as N1 if the tumour is located in the gastric cardia (Thompson, 1997). Distant metastases from oesophageal cancer are most frequently detected in celiac and supraclavicular lymph nodes, liver, lung, and adrenal glands (Quint *et al*, 1995).

Endoscopic ultrasonography (EUS) is often used to determine the depth of tumour invasion and the presence of malignant regional and celiac lymph nodes in patients with oesophageal cancer. Both computed tomography (CT) and ^18^F-fluoro-2-deoxy-D-glucose positron emission tomography (FDG-PET) are commonly applied to determine whether malignant lymph nodes or distant metastases are present.

It is known that EUS, CT, and FDG-PET each have certain limitations. For example, only lymph nodes in the proximity of the oesophageal and gastric wall can be visualised with EUS, as it has a limited penetration depth of approximately 5 cm. As a consequence, metastases in distant lymph nodes or organs can often not be detected by EUS (Kienle *et al*, 2002). Computed tomography is not able to detect metastases in normal-sized lymph nodes. Furthermore, an enlarged lymph node may contain metastases, but can also be enlarged as a consequence of inflammation (Halvorsen and Thompson, 1984; Finch *et al*, 1997; Choi *et al*, 2000). The same is true for abnormal findings in the liver or adrenal glands, for which it is not always clear whether these are metastases or not. Detection of metastases by FDG-PET is based on an altered tissue glucose metabolism. Biochemical changes are known to appear earlier in time than structural changes and also they are more specific (Block *et al*, 1997; Flanagan *et al*, 1997). Nevertheless, lesions less than 1 cm in diameter can be missed by FDG-PET (Lerut *et al*, 2000).

In this study, we evaluated the diagnostic performance of EUS, CT, and FDG-PET as has been reported in the literature. In addition, we determined whether only one or a combination of these investigations should be used in the staging of oesophageal cancer. The focus in this meta-analysis was on the application of EUS for the detection of malignant regional and celiac lymph nodes, the use of CT for the detection of malignant regional and abdominal lymph nodes and distant metastases, and the use of FDG-PET for the detection of malignant regional lymph nodes and distant metastases.

## MATERIALS AND METHODS

### Literature search and data extraction

A PubMed literature search was performed identifying all articles related to the diagnostic use of EUS, CT, and FDG-PET in patients with oesophageal cancer. Search terms that were used to identify such articles were combinations of ‘esophagus’, ‘oesophagus’, ‘cancer’, ‘neoplasm’, ‘carcinoma’, ‘endoscopic ultrasonography’, ‘endosonography’ ‘EUS’, ‘computed tomography’, ‘CT’, ‘positron emission tomography’, and ‘PET’. Abstracts obtained from these searches were evaluated. Articles containing information on the results of EUS, CT, and/or FDG-PET for N and/or M stage of oesophageal cancer and published in the English literature before January 2006 were reviewed. Articles were included if the absolute numbers of true-positive (TP), false-negative (FN), false-positive (FP), and true-negative (TN) test results were available or derivable from the article, which allowed us to construct 2 × 2 contingency tables. The references of articles and reviews, found in the literature search, were also examined to find additional articles that met the inclusion criteria. Studies with potentially overlapping study populations were excluded. For this, we included only the study with the largest patient population and published latest in time, whereas the previous study, with often a smaller subgroup of patients, was excluded. Also excluded were articles published in abstract form only, case reports, editorials, and reviews. In addition, articles containing the results of patients who had undergone prior radiation and/or chemotherapy were excluded if the result of the reference standard could have been influenced by the administration of this treatment. The reference standard was resection, result of fine-needle aspiration (FNA), follow-up with radiographic techniques, and/or clinical follow-up in the article.

Two independent readers (EV, PS) extracted the data from the included articles. The absolute numbers of TP, FN, FP, and TN test results were retrieved or calculated from the published data. Other characteristics that were extracted from each study were origin and publication year of an article, mean age of patients, proportion of males (as percentage of total number of patients), tumour histology, retrospective or prospective set-up of the study, whether or not the patients were consecutively included, whether or not the test results were blindly interpreted, and the reference standard that was used in the study. For articles containing EUS results, the type of EUS probe, whether or not FNA was performed for suspicious lymph nodes, and whether or not dilation was performed in patients with a stenotic tumour were also recorded. From articles containing CT results, information on the type of CT scanner and use of a contrast agent was obtained. From articles containing FDG-PET results, the type of PET scanner was recorded. Inconsistent findings between the two readers were discussed and agreed upon by consensus.

### Statistical analysis

To determine whether publication bias, that is, the selective reporting of manuscripts with more positive results, was present, funnel plots were constructed. A funnel plot is an epidemiologic method for assessing the presence of publication bias. For this, the measure of study size is plotted against the measure of interest. In this study, the measure of study size was the number of patients included in the study, whereas the measure of interest was the natural logarithm of the diagnostic odds ratio (*D*). The idea is that studies with the largest study size will estimate *D* most accurately, whereas studies with a smaller study size will have a more variable result, with both lower and higher values of *D* compared to the larger studies. If this is the case, the plot will have a symmetric, inverted funnel shape. If publication bias is present, the left base of the plot will disappear and the plot is asymmetric and skewed (Terrin *et al*, 2005). Symmetry and shape of the funnel plots were determined by means of visual inspection. To allow assessment of the presence of publication bias by visual inspection, the number of included articles had to be more than 10.

Sensitivities and specificities of EUS, CT, and FDG-PET were pooled using a random effects model. With this method, the variability between studies is taken into account. To estimate the relationship between sensitivities and specificities of each investigation, a random effects summary receiver operating characteristic (SROC) analysis was performed. In an SROC analysis, the logits (log odds) of sensitivity and 1−specificity are subtracted to calculate *D* (*D*=ln(sensitivity/(1−sensitivity))−ln((1−specificity)/specificity)). *D* is the log of the diagnostic odds ratio, which represents a summary measure of the diagnostic performance or discriminatory power of an investigation. *D* ranges from zero to infinity. A value close to zero or far from 1 represents an investigation with good diagnostic performance. The logits are summed to calculate *S* (*S*=ln(sensitivity/(1−sensitivity))+ln((1−specificity)/specificity)), which is a proxy for the positivity criterion of the diagnostic test. When institutions use different thresholds for scoring a test result as positive, different positivity criteria will exist among studies. Subsequently, a linear regression model *D*=*a*+*bS* was estimated, weighted by the inverse of the variance of *D*. We have chosen to perform a weighted regression, as the unweighted regression may not highlight larger studies.

Additional covariates (such as publication year, number of patients, mean age, proportion of males, whether or not the study was performed prospectively, whether or not the patients were included consecutively, and whether or not the test results were interpreted in a blinded fashion) were added to the model to adjust for differences in study and patient characteristics. The effect of a covariate was expressed in the relative diagnostic odds ratio (a value >1, 1, or <1 means superior, equal, or inferior diagnostic performance, respectively) and was considered statistically significant if *P*<0.05. The *meta* and *metareg* commands of STATA 8.0 were used to perform the meta-analysis.

## RESULTS

### Literature search and data extraction

The PubMed literature search for the identification of articles relating to the diagnostic use of EUS in oesophageal cancer patients resulted in 573 hits on the search terms. In total, 31 articles on EUS for regional lymph node metastases (Tio *et al*, 1990; Botet *et al*, 1991; Rice *et al*, 1991; Ziegler *et al*, 1991; Dittler and Siewert, 1993; Grimm *et al*, 1993; Greenberg *et al*, 1994; Yoshikane *et al*, 1994; Binmoeller *et al*, 1995; Hasegawa *et al*, 1996; Hunerbein *et al*, 1996; Natsugoe *et al*, 1996; Pham *et al*, 1998; Vickers, 1998; Bowrey *et al*, 1999; Catalano *et al*, 1999; Nishimaki *et al*, 1999; Salminen *et al*, 1999; Choi *et al*, 2000; Nesje *et al*, 2000; Richards *et al*, 2000; Shinkai *et al*, 2000; Vazquez-Sequeiros *et al*, 2001, 2003; Rasanen *et al*, 2003; Wu *et al*, 2003; Heeren *et al*, 2004; Sihvo *et al*, 2004; DeWitt *et al*, 2005; Lowe *et al*, 2005; Pedrazzani *et al*, 2005) and five articles on EUS for celiac lymph node metastases were included (Binmoeller *et al*, 1995; Catalano *et al*, 1999; Eloubeidi *et al*, 2001; Vazquez-Sequeiros *et al*, 2001; Parmar *et al*, 2002) (Table 1). For CT, the literature search gave 1091 hits. Seventeen articles on CT for regional lymph node metastases met the inclusion criteria (Quint *et al*, 1985; Botet *et al*, 1991; Ziegler *et al*, 1991; Sondenaa *et al*, 1992; Greenberg *et al*, 1994; Yoshikane *et al*, 1994; Flanagan *et al*, 1997; Nishimaki *et al*, 1999; Choi *et al*, 2000; Wren *et al*, 2002; Rasanen *et al*, 2003; Vazquez-Sequeiros *et al*, 2003; Wu *et al*, 2003; Yoon *et al*, 2003; Heeren *et al*, 2004; Sihvo *et al*, 2004; Lowe *et al*, 2005). As some articles containing the results of CT not only reported on celiac lymph node metastases, but also on other abdominal lymph node metastases, we decided to include all articles (five in total) with these results (Quint *et al*, 1985; Becker *et al*, 1986; Watt *et al*, 1989; Van Overhagen *et al*, 1993; Parmar *et al*, 2002). In addition, seven articles on CT for distant metastases were included (Van Overhagen *et al*, 1993; Flamen *et al*, 2000; Wren *et al*, 2002; Rasanen *et al*, 2003; Yoon *et al*, 2003; Sihvo *et al*, 2004; Lowe *et al*, 2005) (Table 2). Our literature search for FDG-PET gave 163 hits. We included 10 articles on FDG-PET for regional lymph node metastases (Flanagan *et al*, 1997; Luketich *et al*, 1997; Choi *et al*, 2000; Lerut *et al*, 2000; Wren *et al*, 2002; Rasanen *et al*, 2003; Yoon *et al*, 2003; Heeren *et al*, 2004; Sihvo *et al*, 2004; Lowe *et al*, 2005). Nine articles were included on FDG-PET for distant metastases (Luketich *et al*, 1997; Flamen *et al*, 2000; Lerut *et al*, 2000; Wren *et al*, 2002; Rasanen *et al*, 2003; Yoon *et al*, 2003; Heeren *et al*, 2004; Sihvo *et al*, 2004; Lowe *et al*, 2005) (Table 3). We found that the interobserver agreement for the data extraction was excellent (*κ*>0.80).

### Regional lymph node metastases

**Figure 1:**
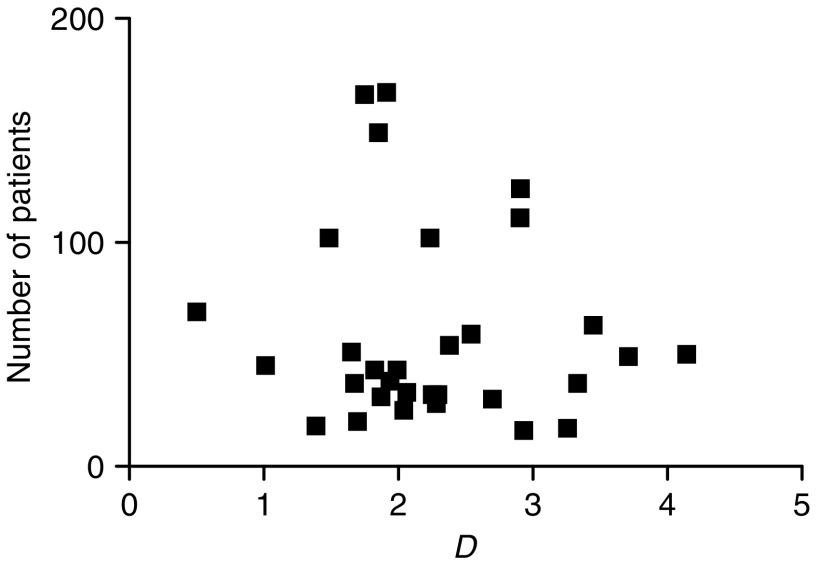
Funnel plot in which the number of patients included in studies on the use of EUS for the detection of regional lymph node metastases was plotted against the log of the diagnostic odds ratio (*D*).

Random effects pooled sensitivity and specificity of EUS for N stage were 0.80 (95% confidence interval (CI) 0.75–0.84) and 0.70 (95% CI 0.65–0.75), respectively, and the pooled log odds ratio was 1.94 (95% CI 1.71–2.17) (Table 4). Visual inspection of the funnel plot revealed that the plot was symmetric (Figure 1), which implies that the presence of publication bias was unlikely. In the present study, no statistically significant differences were found between EUS and EUS-FNA for N stage.

**Figure 2:**
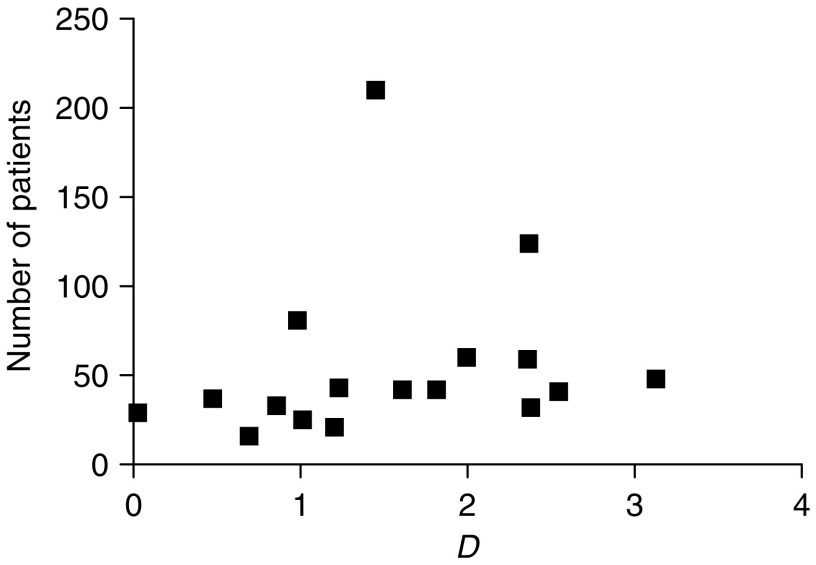
Funnel plot in which the number of patients included in studies on the use of CT for the detection of regional lymph node metastases was plotted against the log of the diagnostic odds ratio (*D*).

Random effects pooled sensitivity and specificity of CT for regional lymph node metastases were 0.50 (95% CI 0.41–0.60) and 0.83 (95% CI 0.77–0.89), respectively. The pooled log odds ratio was 1.40 (95% CI 1.08–1.72) (Table 4). No evidence for publication bias was found (Figure 2).

Random effects pooled sensitivity and specificity of FDG-PET for regional lymph node metastases were 0.57 (95% CI 0.43–0.70) and 0.85 (95% CI 0.76–0.95), respectively. The pooled log odds ratio was 1.71 (95% CI 1.22–2.20) (Table 4). It was not possible to assess whether publication bias was present, as the number of articles was too small (*n*=10).

**Figure 3:**
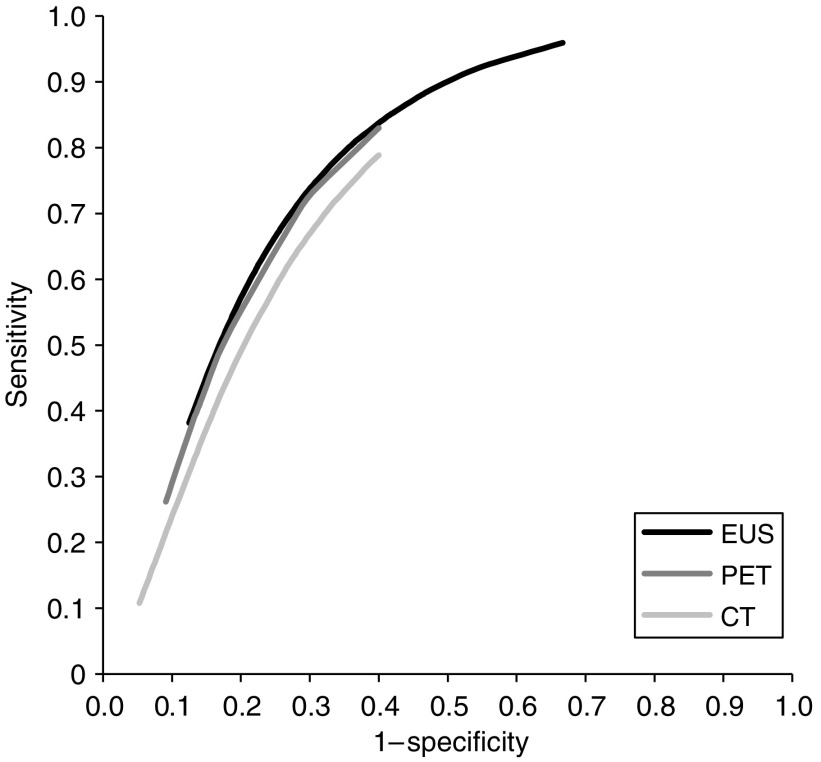
Summary receiver operating characteristic curves for EUS, FDG-PET, and CT for detection of regional lymph node metastases. *P*=not significant.

The estimated SROC curves are shown in Figure 3. The differences between the curves of EUS, CT, and FDG-PET for N staging were not statistically significant. The relative diagnostic odds ratio of CT *vs* EUS was 0.76 (95% CI 0.48–1.21; *P*=0.25) and of FDG-PET *vs* EUS 0.95 (95% CI 0.54–1.67; *P*=0.86). Thus, taking into account the inverse relationship between sensitivity and specificity and different test thresholds across different studies, there were no significant differences in diagnostic performance across these tests for the detection of regional lymph node metastases. Study and patient characteristics also did not show any significant effect on the diagnostic performance of the tests. Nevertheless, comparing the pooled sensitivities and pooled specificities across the tests, statistically significant differences were present, that is, the 95% CI did not always overlap. Sensitivities of CT and FDG-PET for N stage were significantly lower than that of EUS, whereas specificities were significantly higher. This implies that these investigations work at different points on the SROC curve, that is, with EUS being more sensitive and less specific than CT and FDG-PET for regional lymph node metastases, but overall having a similar diagnostic performance (Table 4).

### Celiac and abdominal lymph node metastases

Random effects pooled sensitivity and specificity of EUS for celiac lymph node metastases were 0.85 (95% CI 0.72–0.99) and 0.96 (95% CI 0.92–1.00), respectively, and the pooled log odds ratio was 3.89 (95% CI 2.67–5.11) (Table 4). As the number of articles was only five, assessment of publication bias was not possible.

Random effects pooled sensitivity and specificity of CT for malignant abdominal lymph nodes were 0.42 (95% CI 0.29–0.54) and 0.93 (95% CI 0.86–1.00), respectively. The pooled log odds ratio measured 1.74 (95% CI 0.45–3.04) (Table 4). It was not possible to assess whether publication bias was present.

We have not included an SROC curve for EUS (celiac lymph node metastases) and CT (abdominal lymph node metastases) as we think that these curves could not be fairly compared with each other.

### Distant metastases

Random effects pooled sensitivity and specificity of CT for distant metastases were 0.52 (95% CI 0.33–0.71) and 0.91 (95% CI 0.86–0.96), respectively. The pooled log odds ratio was 2.10 (95% CI 1.59–2.62) (Table 4). The number of articles was too low to assess publication bias.

Random effects pooled sensitivity and specificity of FDG-PET for the detection of distant metastases were 0.71 (95% CI 0.62–0.79) and 0.93 (95% CI 0.89–0.97), respectively. The pooled log odds ratio was 2.93 (95% CI 2.41–3.45) (Table 4). Assessment of publication bias was not possible.

**Figure 4:**
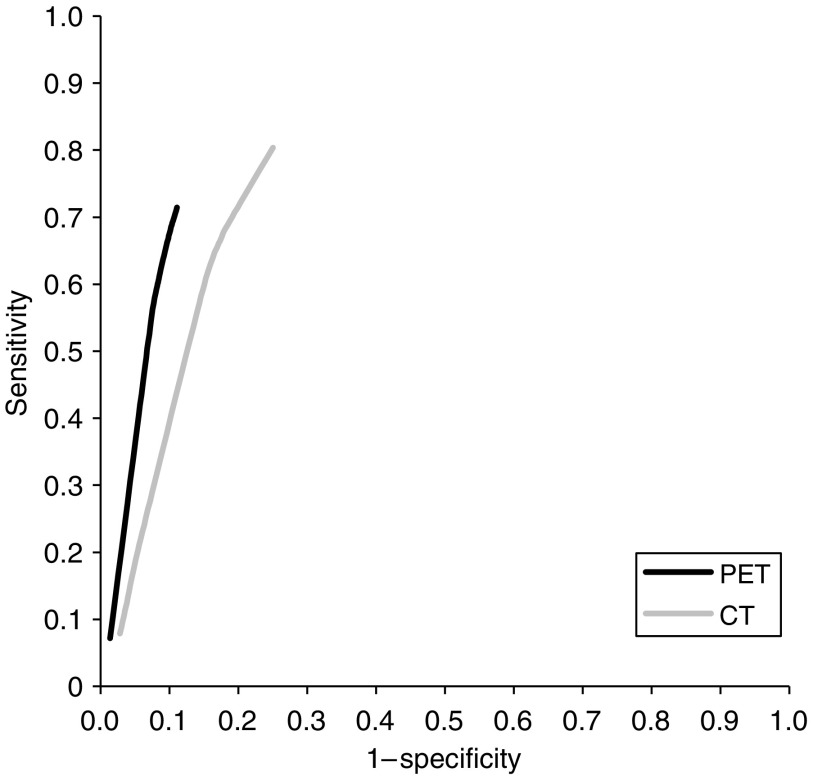
Summary receiver operating characteristic curves for FDG-PET and CT for detection of distant metastases. *P*<0.03.

If the pooled sensitivities, specificities, and log odds ratios across tests were compared separately, we found higher values of FDG-PET for the detection of distant metastases compared to CT, although not statistically significant. Nevertheless, the SROC analysis showed that the diagnostic performance of FDG-PET was significantly higher than the diagnostic performance of CT (relative diagnostic odds ratio=2.26 (95% CI 1.09–4.71), *P*<0.03), taking into account the inverse relationship between sensitivity and specificity and different test thresholds across the studies (Figure 4).

To adjust for differences in study characteristics, various covariates were added in the SROC analysis. This showed that the covariates were not statistically significant (*P*>0.05), and there was significant difference between the diagnostic performance of CT and FDG-PET for the detection of distant metastases.

## DISCUSSION

We performed a meta-analysis to determine the value of EUS, CT, and FDG-PET in the staging of oesophageal cancer patients. We found that EUS was significantly more sensitive but less specific than CT and FDG-PET for the detection of regional lymph node metastases. The overall diagnostic performance of the three tests, however, was similar. Furthermore, we found that the diagnostic performance of FDG-PET was significantly higher than that of CT for distant metastases.

Based on these results, EUS was shown to be particularly useful for the exclusion of regional lymph node metastases. The low number of false-negative results for EUS meant that a negative EUS result will be in most patients a truly negative one. The sensitivities of CT and FDG-PET for the detection of regional lymph node metastases were lower. It is already known that lymph nodes adjacent to the primary oesophageal cancer are difficult to discriminate from the primary tumour with FDG-PET (McAteer *et al*, 1999), which is due to the intense activity in the primary cancer (Flanagan *et al*, 1997) and the limited spatial resolution of PET (Luketich *et al*, 1997; Rankin *et al*, 1998). Low sensitivity of CT for regional lymph node can at least partly be explained by the fact that CT is not able to detect metastases in normal-sized lymph nodes (Choi *et al*, 2000).

Computed tomography and/or FDG-PET can be used to confirm that an enlarged regional lymph node is indeed metastatic, as the number of false-positive results was found to be relatively low for both investigations. However, a better option nowadays is to perform EUS-guided FNA to confirm metastatic disease. This is in line with recommendations in the literature, in which EUS-FNA, CT, and/or FDG-PET are advocated for the exclusion of the presence of regional lymph node metastases in oesophageal cancer patients, particularly if this will affect a treatment decision in these patients. In several studies, it has been demonstrated that the results of EUS-FNA were better than those of EUS alone for determining N stage (Eloubeidi *et al*, 2001; Vazquez-Sequeiros *et al*, 2001; Parmar *et al*, 2002; van Vliet *et al*, 2006), because EUS combined with FNA allowed a cytological differentiation between reactive (non-malignant) and malignant lymph nodes (van Vliet *et al*, 2006).

The presence or absence of regional lymph node metastases has little direct consequences for the treatment decision. For example, patients with a T1–3 tumour without distant metastases and who are fit enough will receive a resection, which is mostly irrespective of the presence of regional lymph node metastases. Nonetheless, it is important to know whether regional lymph node metastases are present if an endoscopic treatment option is considered in patients with early-stage oesophageal cancer. In addition, the presence of regional lymph node metastases plays a role in the comparison of treatment modalities of oesophageal cancer, for example if comparing neoadjuvant therapy *plus* surgery *vs* surgery alone, and has consequences for the prognosis of patients. Therefore, we think that it is important to analyse whether detected lymph nodes are indeed metastatic (by using FNA), particularly if this will affect a treatment decision in patients.

It is also of clinical relevance to determine whether celiac and/or other abdominal lymph node metastases are present. If present, these are considered to be distant metastases (M1b), depending on the localisation of the primary tumour, which could change a treatment decision from a curative to a palliative option. As several articles on CT for staging oesophageal cancer reported not only on celiac lymph nodes, but also on other abdominal lymph nodes, the pooled results of EUS for the detection of celiac lymph node metastases and the pooled results of CT for the detection of abdominal lymph node metastases could not be fairly compared. Nevertheless, as the results of EUS and CT were clearly different, with EUS results being obviously better, it seems likely that EUS is the preferred investigation to determine whether or not celiac lymph node metastases are present in patients with oesophageal cancer. It is important to realise, however, that not all abdominal lymph nodes can be determined with EUS, as the EUS probe has a limited penetration depth of approximately 5 cm. Although no data are available in the literature, we recommend combining EUS with CT to investigate whether, apart from celiac lymph node metastases, other abdominal lymph node metastases are present.

The staging results of EUS for celiac lymph node metastases were even better than those for regional lymph node metastases. An explanation for this could be that only a few studies have reported on the use of EUS for the detection of malignant celiac lymph nodes, which were likely performed in high-volume EUS centres. In contrast, studies that reported EUS results for regional lymph node metastases were performed not only in high-volume centres, but also in low-volume centres. We recently reported that the results of EUS performed in a centre where <50 EUS procedures per endoscopist per year (low-volume centres) are performed were inferior to those from high-volume EUS centres (>50 EUS procedures per endoscopist per year) (van Vliet *et al*, 2006).

Both CT and FDG-PET can be used to detect distant metastases, which determines whether a patient is suitable for a curative treatment option. The reported results of the investigations varied widely (Table 2 and 3) and it is currently difficult to determine how these investigations should be used during staging of patients with oesophageal cancer. The pooled sensitivity of CT for the detection of distant metastases was lower than that of FDG-PET, whereas specificity was equivalent. The results for FDG-PET were comparable to the results found in a previous meta-analysis on FDG-PET for this indication (van Westreenen *et al*, 2004). In our SROC analysis, we found that the diagnostic performance of FDG-PET was significantly better than that of CT (Figure 4). This method takes both sensitivity and specificity into account and adjusts for potential differences in test thresholds across studies. Two studies in which FDG-PET was directly compared with CT for the detection of distant metastases (Luketich *et al*, 1999; Flamen *et al*, 2000) demonstrated that both sensitivity and specificity of FDG-PET for distant metastases were higher compared to those of CT. In contrast, other studies comparing FDG-PET with CT found similar accuracies for both investigations for the detection of distant metastases (Wren *et al*, 2002; Kneist *et al*, 2003, 2004; Rasanen *et al*, 2003; Sihvo *et al*, 2004).

Several publications have reported on the additional value of FDG-PET for the detection of distant metastases, and it has been demonstrated that distant metastases were detected with FDG-PET in 0–20% of patients with oesophageal cancer, which were not found with other investigations. In patients in whom distant metastases were detected with FDG-PET, the treatment plan was corrected from a curative to a palliative option and unnecessary surgery was precluded (Block *et al*, 1997; Flanagan *et al*, 1997; Kole *et al*, 1998; Rankin *et al*, 1998; Luketich *et al*, 1999; Flamen *et al*, 2000; Lerut *et al*, 2000; Wren *et al*, 2002; Imdahl *et al*, 2004; van Westreenen *et al*, 2005). Disadvantages of FDG-PET include the high costs and the fact that it mostly needs to be combined with CT to localise a lesion that is visualised by FDG-PET. It remains to be established whether the costs of FDG-PET are compensated for by the cost reduction of resections that are prevented due to the finding of additional metastases with FDG-PET. In the early days of FDG-PET, it was unclear whether FDG-PET should be the initial investigation to detect metastases in oesophageal cancer, or, alternatively, whether FDG-PET should be performed if EUS and CT have already been performed. The performance of FDG-PET alone does not seem to be the ideal method for the detection of distant metastases, but the combined use of FDG-PET and CT could well be of significance. This is already on the horizon, as integrated FDG-PET/CT machines are increasingly becoming available. Unfortunately, the value of PET-CT could not be clearly determined by our meta-analysis, as there were not enough well-performed studies available.

Our meta-analysis has some limitations. First, abstracts were assessed to identify articles reporting on the results of EUS, CT, and FDG-PET. There is a small risk that some articles of which the abstract revealed that it was highly unlikely that the article contained information on the results of EUS, CT, and/or FDG-PET were excluded from evaluation. It is also possible that articles with results on EUS, CT, and FDG-PET did not match the search terms. To correct for this, references of included articles and reviews were examined for additional articles that met the inclusion criteria.

Second, only studies with the results of EUS, CT, and/or FDG-PET for the detection of lymph node or distant metastases from which absolute numbers of TP, FN, FP, and TN test results were available or derivable were included, which is advocated in the STARD guidelines (Bossuyt *et al*, 2003). Several studies did not report these absolute numbers precluding thorough statistical evaluation. In addition, some of our analyses were based on limited numbers of publications and it could therefore be that some differences in this meta-analysis were not statistically significant due to these limited sample sizes.

Third, it seems likely that verification bias played a role in all articles included in our meta-analysis, because the results of staging investigations performed in patients with oesophageal cancer were used to decide whether or not FNA should be performed and whether or not patients could undergo a resection. Funnel plots, as shown in Figure 1 and 2, revealed that publication bias was probably not present in the meta-analysis regarding EUS and CT for the detection of regional lymph node metastases. Nevertheless, for the other analyses, the number of included studies was too small to evaluate publication bias.

Fourth, the time period in which the evaluated articles were published might also be a factor to consider. The initial reported results of a new investigation are often more favorable than those published later in time if the procedure is used in a less selected study population. We accounted for this potential effect by including the year of publication as a covariate in our analysis. The results showed that the publication year was not a significant predictor of diagnostic performance in our study.

Fifth, the methodological quality of some studies included in this meta-analysis was limited. For example, several studies that are shown in Table 1, 2, 3 were retrospectively performed and in some studies the test results were not blindly interpreted. In addition, missing values were present for some covariates, particularly for the covariates inclusion (consecutive or not) and interpretation (blinded or not).

Sixth, only a few articles on CT and EUS reported nodal cutoff sizes for positivity. For this reason, we could not perform a sub-analysis to determine which nodal cutoff size was most accurate. In addition, only a few articles reported on the location of the tumour and, therefore, it was not possible to determine whether the results of EUS differed for the various locations of the tumours, that is, cervical, upper 1/3 thoracic, central 1/3 thoracic, lower 1/3 thoracic, and gastrooesophageal junction. In addition, it would have been clinically interesting if the localisation of the primary tumour could have been combined with nodal status. The same was true for the location of distant metastases, which was also not reported in the majority of studies and, if reported, results were not given per location. Therefore, it was not possible to calculate results for the different metastatic locations in our meta-analysis.

Finally, it is known that experience is an important factor in performing EUS. Nevertheless, in the majority of articles, it was not reported how experienced endoscopists were and, therefore, we could not determine whether EUS results varied for different levels of experience. Moreover, no information was given on the number of patients with squamous cell carcinoma or adenocarcinoma in several studies and, if reported, results were mostly not reported per tumour histology.

In conclusion, the presence of malignant regional lymph nodes can be determined with EUS, CT, and FDG-PET. Of these, EUS has the highest sensitivity but also the lowest specificity for regional lymph node metastases. To exclude the presence of regional lymph node metastases, EUS should be used, whereas detected lesions should be confirmed with FNA, or, alternatively, with CT and/or FDG-PET, particularly if this will affect a treatment decision in patients. Both EUS and CT should probably be performed to determine whether abdominal lymph node metastases are present, with EUS being highly sensitive for celiac lymph node metastases and CT being performed to detect other abdominal lymph node metastases. Both CT and FDG-PET can be used to detect the presence of distant metastases; however, the results suggest that FDG-PET has a higher diagnostic performance than CT. Nonetheless, it seems likely that its combined use could be of clinical value, with FDG-PET detecting possible metastases and CT confirming or excluding their presence and precisely determining the location(s). Nowadays, integrated FDG-PET/CT machines are increasingly being available and their use has become more common.

## Figures and Tables

**Table 1:** Characteristics of studies containing absolute numbers of TP, FN, FP, and TN test results of EUS for regional and celiac lymph node metastases

**Study**	**Study type**	**Blinded interpretation of test results**	**Resection as reference standard**	**FNA as reference standard**	**Other reference standard**	**Number of patients**	**Sensitivity (%)**	**Specificity (%)**
*Regional lymph node metastases*								
Tio *et al* (1990)	Not reported	Not reported	Yes	No		111	73/77 (95)	17/34 (50)
Ziegler *et al* (1991)	Prospective	Not reported	Yes	No	Autopsy	37	16/25 (64)	9/12 (75)
Rice *et al* (1991)	Not reported	Not reported	Yes	No		20	7/10 (70)	7/10 (70)
Botet *et al* (1991)	Prospective	Not reported	Yes	No		50	35/36 (97)	9/14 (64)
Grimm *et al* (1993)	Prospective	Not reported	Yes	No		63	37/41 (90)	17/22 (77)
Dittler and Siewert (1993)	Not reported	Not reported	Yes	No		167	85/114 (75)	37/53 (70)
Yoshikane *et al* (1994)	Not reported	Yes	Yes	No		25	7/12 (58)	11/13 (85)
Greenberg *et al* (1994)	Prospective	Not reported	Yes	No		16	6/10 (60)	6/6 (100)
Binmoeller *et al* (1995)	Prospective	Not reported	Yes	No		38	26/29 (90)	4/9 (44)
Hasegawa *et al* (1996)	Not reported	Not reported	Yes	No		18	4/8 (50)	8/10 (80)
Natsugoe *et al* (1996)	Prospective	Not reported	Yes	No		37	4/5 (80)	28/32 (88)
Hunerbein *et al* (1996)	Prospective	Not reported	Yes	No		17	13/14 (93)	2/3 (67)
Pham *et al* (1998)	Prospective	Yes	Yes	No		28	14/16 (88)	7/12 (58)
Vickers, 1998	Prospective	Not reported	Yes	No		49	35/36 (97)	7/13 (54)
Bowrey *et al* (1999)	Not reported	Not reported	Yes	No		30	17/19 (89)	7/11 (64)
Salminen *et al* (1999)	Prospective	Yes	Yes	No		32	19/20 (95)	4/12 (33)
Catalano *et al* (1999)	Prospective	Not reported	Yes	Yes		149	75/95 (79)	34/54 (63)
Nishimaki *et al* (1999)	Prospective	Not reported	Yes	No		166	88/110 (80)	33/56 (59)
Shinkai *et al* (2000)	Not reported	Not reported	Yes	No		102	41/54 (76)	28/48 (58)
Nesje *et al* (2000)	Prospective	Not reported	Yes	No		54	36/46 (78)	6/8 (75)
Richards *et al* (2000)	Retrospective	Not reported	Yes	No		69	19/42 (45)	18/27 (67)
Choi *et al* (2000)	Prospective	Yes	Yes	No		45	15/30 (50)	11/15 (73)
Vazquez-Sequeiros *et al* (2001)	Retrospective	Not reported	Yes	No		33	14/22 (64)	9/11 (82)
Vazquez-Sequeiros *et al* (2003)	Prospective	Yes	Yes	Yes		124	68/85 (80)	32/39 (82)
Wu *et al* (2003)	Not reported	No	Yes	No		31	13/19 (68)	9/12 (75)
Rasanen *et al* (2003)	Prospective	Not reported	Yes	No	Follow-up	32	17/19 (89)	7/13 (54)
Heeren *et al* (2004)	Not reported	Yes	Yes	Yes	Follow-up	43	18/26 (69)	13/17 (76)
Sihvo *et al* (2004)	Prospective	No	Yes	No		43	22/26 (85)	9/17 (53)
Lowe *et al* (2005)	Prospective	Yes	Yes	Yes		59	38/44 (86)	10/15 (67)
Pedrazzani *et al* (2005)	Retrospective	Not reported	Yes	No		51	25/37 (68)	10/14 (71)
DeWitt *et al* (2005)	Retrospective	Not reported	Yes	No		102	48/66 (73)	28/36 (78)
								
*Celiac lymph node metastases*								
Binmoeller *et al* (1995)	Prospective	Not reported	Yes	No		35	3/4 (75)	29/31 (94)
Catalano *et al* (1999)	Prospective	Not reported	Yes	Yes		149	19/23 (83)	124/126 (98)
Eloubeidi *et al* (2001)	Retrospective	No	Yes	Yes		102	48/62 (77)	34/40 (85)
Vazquez-Sequeiros *et al* (2001)	Retrospective	Not reported	Yes	No		33	3/4 (75)	29/29 (100)
Parmar *et al* (2002)	Retrospective	Not reported	Yes	Yes		20	18/18 (100)	1/2 (50)

TP=true-positive; FN=false-negative; FP=false-positive; TN=true-negative; EUS=endoscopic ultrasonography; FNA=fine-needle aspiration.

**Table 2:** Characteristics of studies containing absolute numbers of TP, FN, FP, and TN test results of CT for regional lymph node metastases, abdominal lymph node metastases, and distant metastases

**Study**	**Study type**	**Blinded interpretation of test results**	**Resection as reference standard**	**FNA as reference standard**	**Other reference standard**	**Number of patients**	**Sensitivity (%)**	**Specificity (%)**
*Regional lymph node metastases*								
Quint *et al* (1985)	Retrospective	Not reported	Yes	No		33	11/18 (61)	9/15 (60)
Ziegler *et al* (1991)	Prospective	Not reported	Yes	No	Autopsy	37	10/24 (42)	9/13 (69)
Botet *et al* (1991)	Prospective	Not reported	Yes	No		42	23/29 (79)	8/13 (62)
Sondenaa *et al* (1992)	Retrospective	Not reported	Yes	No		42	5/23 (22)	18/19 (95)
Yoshikane *et al* (1994)	Not reported	Yes	Yes	No		25	4/12 (33)	11/13 (85)
Greenberg *et al* (1994)	Prospective	Not reported	Yes	No		16	5/10 (50)	4/6 (67)
Flanagan *et al* (1997)	Retrospective	Yes	Yes	No		29	5/18 (28)	8/11 (73)
Nishimaki *et al* (1999)	Prospective	Not reported	Yes	No		210	81/136 (60)	55/74 (74)
Choi *et al* (2000)	Prospective	Yes	Yes	No		48	13/32 (41)	16/16 (100)
Wren *et al* (2002)	Retrospective	Not reported	Yes	Yes	Autopsy/follow-up	21	4/7 (57)	10/14 (71)
Vazquez-Sequeiros *et al* (2003)	Prospective	Yes	Yes	Yes		124	40/85 (47)	36/39 (92)
Wu *et al* (2003)	Not reported	No	Yes	No		41	17/22 (77)	15/19 (79)
Yoon *et al* (2003)	Prospective	Yes	Yes	No	Follow-up	81	12/39 (31)	36/42 (86)
Rasanen *et al* (2003)	Prospective	Not reported	Yes	No	Follow-up	32	9/19 (47)	12/13 (92)
Heeren *et al* (2004)	Not reported	Yes	Yes	Yes	Follow-up	60	17/39 (44)	19/21 (90)
Sihvo *et al* (2004)	Prospective	No	Yes	No		43	11/26 (42)	14/17 (82)
Lowe *et al* (2005)	Prospective	Yes	Yes	Yes		59	37/44 (84)	10/15 (67)
								
*Abdominal lymph node metastases*								
Quint *et al* (1985)	Retrospective	Not reported	Yes	No		33	2/3 (67)	26/30 (87)
Becker *et al* (1986)	Retrospective	Yes	Yes	No		50	13/23 (57)	27/27 (100)
Watt *et al* (1989)	Prospective	Yes	Yes	No		65	11/35 (31)	26/30 (87)
Van Overhagen *et al* (1993)	Prospective	Yes	Yes	Yes		86	13/27 (48)	55/59 (93)
Parmar *et al* (2002)	Retrospective	Not reported	Yes	Yes		20	5/18 (28)	1/2 (50)
								
*Distant metastases*								
Van Overhagen *et al* (1993)	Prospective	Yes	Yes	Yes		113	38/54 (70)	50/59 (85)
Flamen *et al* (2000)	Prospective	Yes	Yes	No	Follow-up	74	14/34 (41)	33/40 (83)
Wren *et al* (2002)	Retrospective	Not reported	Yes	Yes	Autopsy/follow-up	24	10/12 (83)	9/12 (75)
Rasanen *et al* (2003	Prospective	Not reported	Yes	No	Follow-up	42	5/15 (33)	26/27 (96)
Yoon *et al* (2003)	Prospective	Yes	Yes	No	Follow-up	81	1/7 (14)	70/74 (95)
Sihvo *et al* (2004)	Prospective	No	Yes	No		55	6/19 (32)	35/36 (97)
Lowe *et al* (2005)	Prospective	Yes	Yes	Yes		48	21/26 (81)	18/22 (82)

TP=true-positive; FN=false-negative; FP=false-positive; TN=true-negative; CT=computed tomography; FNA=fine-needle aspiration.

**Table 3:** Characteristics of studies containing absolute numbers of TP, FN, FP, and TN test results of FDG-PET for regional lymph node metastases and distant metastases

**Study**	**Study type**	**Blinded interpretation of test results**	**Resection as reference standard**	**FNA as reference standard**	**Other reference standard**	**Number of patients**	**Sensitivity (%)**	**Specificity (%)**
*Regional lymph node metastases*								
Luketich *et al* (1997)	Retrospective	Not reported	No	Yes	Follow-up	21	9/20 (45)	1/1 (100)
Flanagan *et al* (1997)	Retrospective	Yes	Yes	No		29	13/18 (72)	9/11 (82)
Lerut *et al* (2000)	Prospective	Not reported	Yes	Yes		29	4/18 (22)	10/11 (91)
Choi *et al* (2000)	Prospective	Yes	Yes	No		48	26/32 (81)	14/16 (88)
Wren *et al* (2002)	Retrospective	Not reported	Yes	Yes	Autopsy/follow-up	21	5/7 (71)	12/14 (86)
Yoon *et al* (2003)	Prospective	Yes	Yes	No	Follow-up	81	25/39 (64)	29/42 (69)
Rasanen *et al* (2003)	Prospective	Not reported	Yes	No	Follow-up	32	7/19 (37)	13/13 (100)
Heeren *et al* (2004)	Not reported	Yes	Yes	Yes	Follow-up	61	22/40 (55)	15/21 (71)
Sihvo *et al* (2004)	Prospective	No	Yes	No		43	9/26 (35)	17/17 (100)
Lowe *et al* (2005)	Prospective	Yes	Yes	Yes		59	36/44 (82)	9/15 (60)
								
*Distant metastases*								
Luketich *et al* (1997)	Retrospective	Not reported	No	Yes	Follow-up	35	7/8 (88)	25/27 (93)
Lerut *et al* (2000)	Prospective	Not reported	Yes	Yes		42	10/13 (77)	26/29 (90)
Flamen *et al* (2000)	Prospective	Yes	Yes	No	Follow-up	74	25/34 (74)	36/40 (90)
Wren *et al* (2002)	Retrospective	Not reported	Yes	Yes	Autopsy/follow-up	24	8/12 (67)	11/12 (92)
Yoon *et al* (2003)	Prospective	Yes	Yes	No	Follow-up	81	3/7 (43)	73/74 (99)
Rasanen *et al* (2003)	Prospective	Not reported	Yes	No	Follow-up	42	7/15 (47)	24/27 (89)
Sihvo *et al* (2004)	Prospective	No	Yes	No		55	10/19 (53)	32/36 (89)
Heeren *et al* (2004)	Not reported	Yes	Yes	Yes	Follow-up	74	21/27 (78)	43/47 (91)
Lowe *et al* (2005)	Prospective	Yes	Yes	Yes		48	21/26 (81)	20/22 (91)

TP=true-positive; FN=false-negative; FP=false-positive; TN=true-negative; FDG=^18^F-fluoro-2-deoxy-D-glucose positron emission tomography; FNA=fine-needle aspiration.

**Table 4:** Summary of the number of included studies, the total number of patients, pooled sensitivity, pooled specificity, and the pooled log odds ratio given per disease and investigation

**Disease**	**Investigation**	**Number of included studies**	**Total number of patients**	**Pooled sensitivity (95% CI)**	**Pooled specificity (95% CI)**	**Pooled log odds ratio (95% CI)**
Regional lymph node metastases	EUS	31	1841	0.80 (0.75–0.84)	0.70 (0.65–0.75	1.94 (1.71–2.17)
Regional lymph node metastases	CT	17	943	0.50 (0.41–0.60)	0.83 (0.77–0.89)	1.40 (1.08–1.72)
Regional lymph node metastases	FDG-PET	10	424	0.57 (0.43–0.70)	0.85 (0.76–0.95)	1.71 (1.22–2.20)
Celiac lymph node metastases	EUS	5	339	0.85 (0.72–0.99)	0.96 (0.92–1.00)	3.89 (2.67–5.11)
Abdominal lymph node metastases	CT	5	254	0.42 (0.29–0.54)	0.93 (0.86–1.00)	1.74 (0.45–3.04)
Distant metastases	CT	7	437	0.52 (0.33–0.71)	0.91 (0.86–0.96)	2.10 (1.59–2.62)
Distant metastases	FDG-PET	9	475	0.71 (0.62–0.79)	0.93 (0.89–0.97)	2.93 (2.41–3.45)

CI=confidence interval; EUS=endoscopic ultrasonography; CT=computed tomography; FDG=^18^F-fluoro-2-deoxy-D-glucose positron emission tomography.
